# Bilateral total knee replacement for hemophilic arthropathy: a case report

**DOI:** 10.1186/s12891-025-08611-1

**Published:** 2025-04-25

**Authors:** Mahmoud A. Hafez, Mohamed Talaat, Ahmad Yaman Kebbe, Abdelrahman Helmy, Abdelrahman M Makram

**Affiliations:** 1 The Orthopaedic Department, Faculty of Medicine, October 6 University, Giza, Egypt; 2 Department of Hematology, Faculty of Medicine, October 6 University, Giza, Egypt; 3 Orthopaedics & Trauma Surgery, Gelsenkirchen, Nordrheinwestfalen, Germany; 4 Department of Obstetrics and Gynecology, Stadtkrankenhaus Korbach, 34497 Korbach, Hesse, Germany

**Keywords:** Von Willebrand disease, Total knee replacement, Patient-specific templating, Valgus deformity, Fused knee arthroplasty

## Abstract

Von Willebrand disease (vWD) and hemophilia present unique challenges in orthopedic surgery due to their inherent bleeding risks and potential complications. While joint bleedings are well-documented in hemophilia, they are less understood in vWD, especially regarding their impact on daily activities and the development of arthropathy. We present a case of a young female patient aged 25 years with vWD Type 3 who underwent bilateral total knee replacement (TKR) due to end-stage knee osteoarthritis. Following two previous operations of the right knee at the age of 14, the patient had high degree of extra-articular valgus and intra-articular deformity, ankylosed knee in 30^o^ flexion, osteopenia, and chronic patellar dislocation. Bilateral TKR was performed using patient-specific templating (PST) technology, which includes preoperative planning using a CT scan followed by surgical execution. Financial constraints and limited prosthetic resources necessitated a sequential approach to TKR, addressing the more affected right knee first. Despite many challenges, successful outcomes were achieved, with significant improvements in pain, mobility, overall satisfaction, and no complications at two-year follow-up. Our case highlights the importance of individualized treatment strategies, meticulous surgical planning, and postoperative care in managing knee arthropathy patients with bleeding disorders.

**Supplementary Information:**

The online version contains supplementary material available at 10.1186/s12891-025-08611-1.

## Introduction

Von Willebrand disease (vWD) is a congenital bleeding disorder that affects approximately 0.6–1.3% of the population [1]. The etiology of vWD is rooted in the deficiency (Type 1), dysfunction (Type 2), or virtually near absence (Type 3) of von Willebrand factor (vWF) [2], predominantly manifesting by mucocutaneous bleeding and menorrhagia [3]. Despite being the most prevalent inherited bleeding disorder, vWD remains underdiagnosed, particularly in low-and-middle-income countries, where access to diagnostic tools and optimal treatment remains a significant challenge. The 2022 World Federation of Hemophilia Annual Global Survey reported that only 103,844 individuals have been diagnosed with vWD worldwide, with most cases still receiving suboptimal treatment due to limited access to replacement therapies or prophylaxis, which are now considered global standards of care [4]. This lack of equitable access to care may contribute to the underreporting of joint-related complications in vWD, further limiting our understanding of its impact on long-term musculoskeletal health.

While hemarthrosis is well-documented in hemophilia patients (those lacking coagulation factors VIII, IX, or XI) [5, 6], data on the implications of vWD on joint replacement is lacking. Still, joint bleeding is recognized in vWD; however, it is more common in severe cases, especially those with low Factor VIII levels. Around half of Type 3 vWD patients and 5–10% of moderate to severe Type 1 and Type 2 vWD patients experience joint bleeding. However, there is a significant lack of data on the presence and severity of blood-induced arthropathy in vWD [7] or the general impact of joint health on daily activities [8].

To control vWD-induced knee arthropathy, some drugs like desmopressin can be suggested along with health education to reduce specific activities that put extra strain on the knees like squatting. Contrastingly, for individuals experiencing advanced painful hemarthropathy of the knee, total knee replacement (TKR) serves as the final option [6–8]. It is worth noting that undergoing simultaneous bilateral TKR (simBTKR) is not recommended for those patients due to the notable risk of heightened bleeding during and after the surgical procedure [9].

In this paper, we aim to present the clinical outcomes of bilateral TKR using patient-specific templating (PST) on a patient with bilateral knee osteoarthritis suffering from vWD Type 3.

## Case presentation

**Fig. 1:**
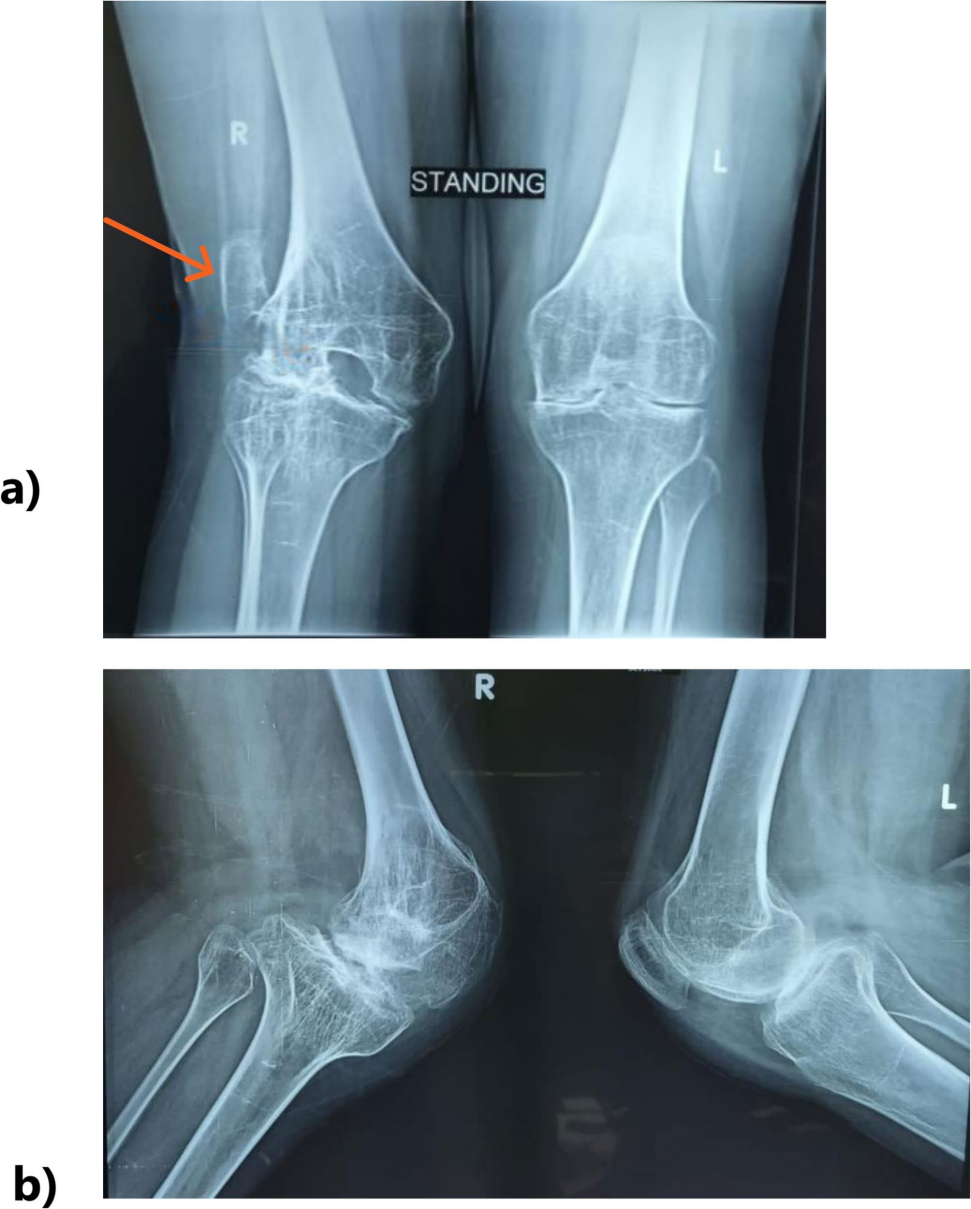
**a)** Preoperative images displaying an x-ray featuring the anterior-posterior (AP) view of both knees, illustrating on the right knee the extra-articular valgus deformity, severe osteopenia, and lateral patellar dislocation (orange arrow) **b)** Preoperative images displaying an x-ray showing lateral view of both knees. The right patella is not located anteriorly and not seen in this view

A 25-year-old female presented to our setting with a recent fall, bilateral severe knee osteoarthritis, osteopenia, and severe extra-articular valgus deformity of 35^o^, intra-articular deformity, ankylosed knees in 30^o^ flexion deformity, and chronic patellar dislocation of the right knee. History-taking revealed that she was diagnosed with vWD Type 3 at the age of one year, with no family history of the condition. From her documents, she had plasma vWF antigen levels (< 10 IU/dL), Ristocetin cofactor activity (< 10 IU/dL), and Factor VIII levels (5 IU/dL) at the time of diagnosis. She had no other comorbidities or significant family history. At the age of 14, a traumatic injury to the right knee resulted in hematomas, causing a fixed flexion deformity at 30° to develop within two months, which led to osteoarthritis and genu valgus of the right knee. Thus, she underwent corrective osteotomy with limited relief (Fig. 1a-b). Hence, the ultimate solution was bilateral TKR.

The patient underwent a patient-specific approach to manage the condition. In both knee surgeries, a tourniquet was employed intraoperatively for hemostasis. Despite the hematologist (MT) recommending simBTKR using PST to reduce the risk of bleeding and operative time from two separate operations [10–14], a sequential approach was adopted due to financial and prosthetic availability constraints. The right knee was operated on first given its more advanced status, followed by the left knee one year later.

To perform the PST technique, preoperative CT scanning was performed to make the necessary measurements for the 3D printing of the cutting guides [11].

During the hospital stay, the patient received Sulfasalazine 1 g, and after discharge, Linezolid 600 mg was prescribed as part of the medication regimen to manage postoperative care and address any associated concerns.

Meticulous hemostatic techniques were employed throughout the surgery. Severe osteoporosis was noted in the bone. For both knees, NexGen Legacy Knee components (Zimmer, Warsaw, Indiana, U.S.) were utilized. Additional procedures included patellar tendon plication and thorough irrigation. Despite the potential complications of vWD Type 3, both knees underwent successful TKR without complications. Postoperatively, structured physiotherapy sessions were performed to facilitate rehabilitation and restore knee functionality.

A hemostatic protocol was designed and implemented as follows (Table 1):

- Preoperatively, the patient received tranexamic acid 500 mg intravenously t.i.d for one day. Moreover, thirty minutes before surgery, the patient received 5000 IU (80 IU/Kg) of Octanate and received a shot of 500 mg tranexamic acid. Octanate was used as it is the most accessible and cost-effective option available in our setting. Moreover, the dosage of the Octanate was above the recommended 50 IU/kg/day to ensure less bleeding.
- Intraoperatively, the patient received an IV infusion of 1500 mg tranexamic acid over two hours during surgery as well as two units of fresh frozen plasma (10 mL/Kg).
- Postoperatively, a drain was inserted to facilitate early detection of bleeding. Moreover, the patient received another 5000 IU Octanate repeated and intravenous 150 mg tranexamic acid once per day for three days. The Octanate dosage was tapered to 3000 IU once per day till reaching two weeks postoperatively. Subsequent tapering to 1500 IU was done for an additional month following the initial two weeks.

**Table 1:** The total pre-, intra-, and postoperative doses of octanate, Tranexamic acid, and fresh frozen plasma used for each procedure

	Preoperative	Intraoperative	Postoperative	Total
**Octanate**	5000 IU	Nil	5000 IU x 6 + 3000 IU x 11 + 1500 IU x 30	113,000 IU
**Tranexamic acid**	500 mg x 4	500 mg x 3	150 mg x 3	3950 mg
**Fresh frozen plasma**	Nil	250 mL x 2	Nil	500 mL

**Fig. 2:**
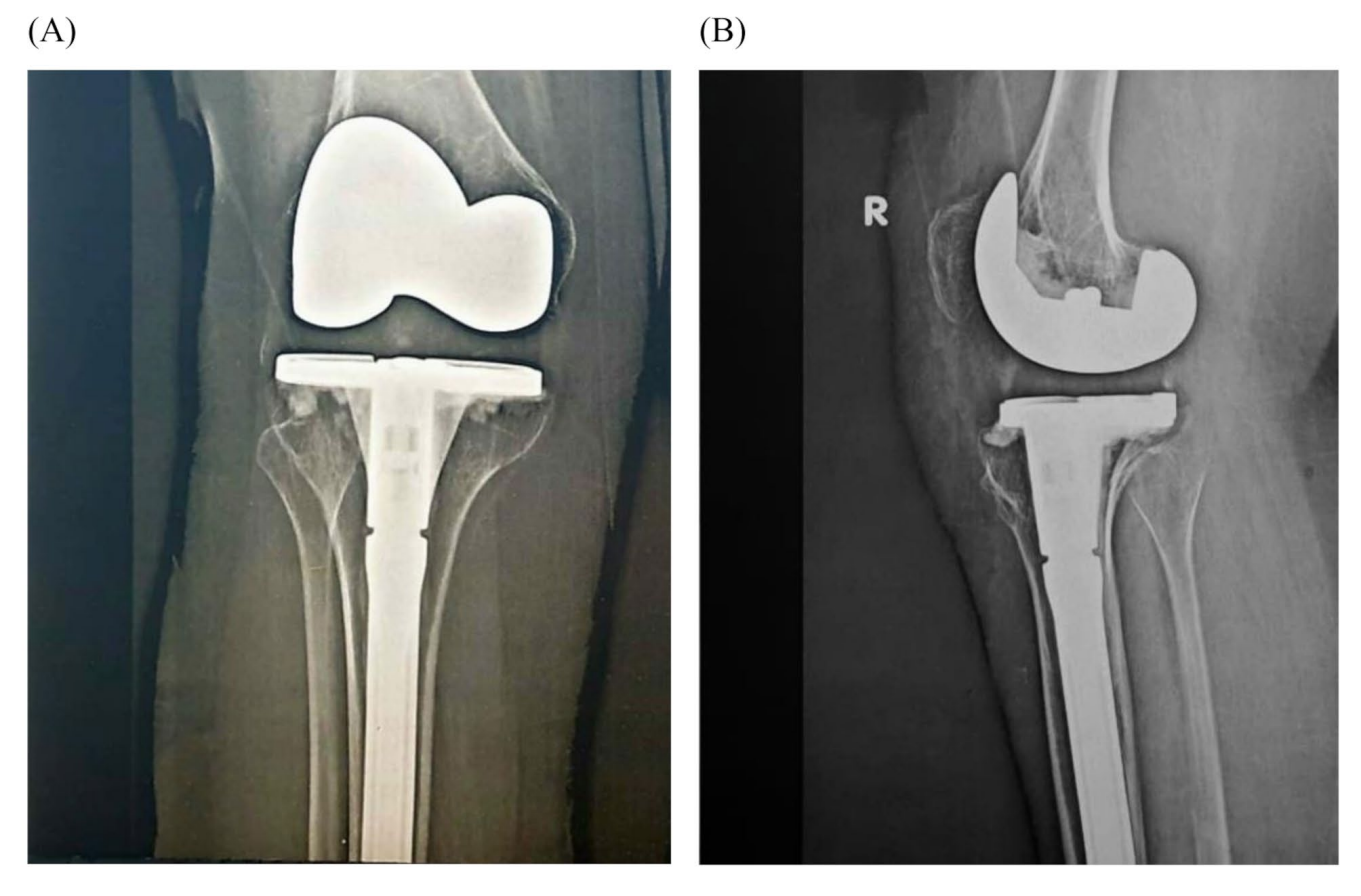
Postoperative antero-posterior (**A**) and lateral (**B**) x-rays of the right knee

**Fig. 3:**
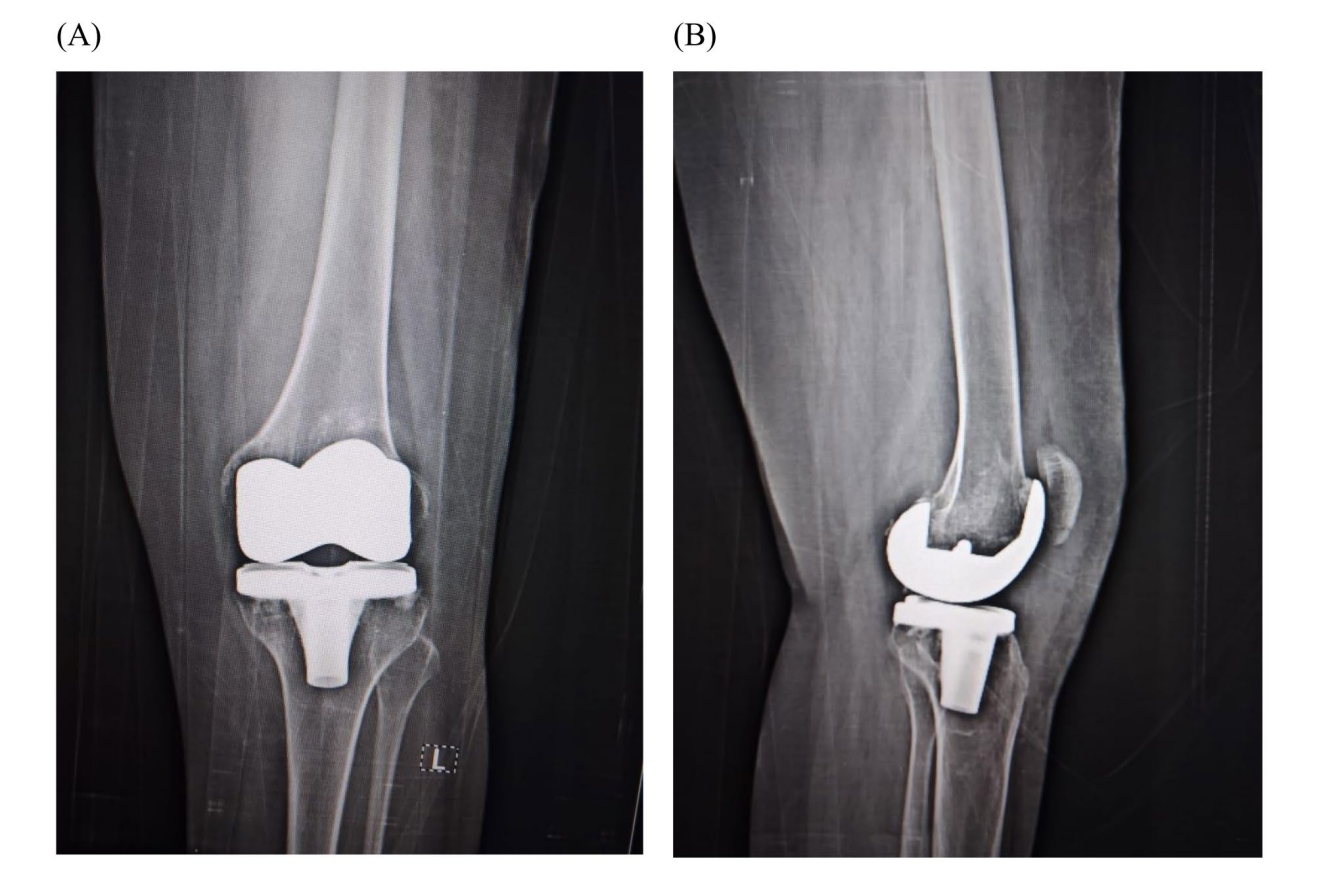
Postoperative antero-posterior (**A**) and lateral (**B**) x-rays of the left knee

Following both operations, a drop in hemoglobin levels was observed, namely from 13.1 g/dL to 9.4 g/dL in the first surgery and from 12.5 g/dL to 9.8 g/dL in the second one. In both instances, the drain collected around 20 mL on the first postoperative day and nearly 0 mL by the fourth day. The patient demonstrated satisfactory progress during follow-up visits with significant improvement in pain and mobility in both knees. At the last follow-up appointment (24 and 12 months for the right and left knee, respectively), the patient had no complaints and still maintained good functionality of the prosthetic knee joint (Figs. 2 and 3). The knee society score (KSS) preoperatively was 22 and 31 for the right and left knees, respectively. The KSS improved to 88 and 89, respectively, at the 12-month follow-up. The patient had a pain assessment of 6/10 in both knees preoperatively, which has improved to 2/10 postoperatively

## Discussion

A case of vWD Type 3 requiring bilateral TKR is presented in this paper. The operation was performed using PST. A sequential approach was chosen over a simultaneous one due to the financial constraints faced by the patient and the limited availability of prosthetic resources. Both procedures were uneventful, and the patient had significant improvement in pain scale, functional knee score, and overall health. The remarkably low blood loss observed during surgery was achieved through the combined effects of PST, which optimized preoperative planning to reduce surgical time and minimize unnecessary soft tissue handling; tranexamic acid, which stabilized clot formation and reduced fibrinolysis; and meticulous surgical technique, including careful tissue handling and the strategic use of a tourniquet. This reflects the case use of a simple, cheap hematological protocol for vWD patients.

Hemarthropathic knees pose a challenge when performing TKR due to the presence of chronic synovitis. For severe cases, a three-stage TKR can be employed, which encompasses correction and lengthening of the femur using Taylor spatial flame, then conversion from external to internal fixation, and lastly the TKR itself [15]. Using computer-assisted orthopedic surgery like patient-specific instrumentation, robotics, and navigation can provide an extra advantage in those cases as they may decrease blood loss, help accommodate extra-articular deformities, and prevent the surgeon from accessing the intramedullary femoral canal [14, 16].

Patients with vWD are perceived to face a fruitful of hurdles when they are in need for a surgical intervention [17]. Those patients when undergoing major surgery typically receive bleeding prophylaxis using the deficient factor. However, some studies have reported increased postoperative thrombotic events [18]. This is possibly due to the evidenced surge in vWF and factor VIII under stress conditions like surgery and inflammation [19–22]. One study has found that vWD patients undergoing major surgery and receiving fondaparinux for the prevention of major thrombotic events, did not experience major bleeding nor required packed RBC transfusion [17]. Still, patients with vWD have more blood loss during primary or revision total knee or hip replacement [23] and require more packed RBC transfusion [24]. Nevertheless, the management of those patients does not only depend on good hemostatic measures, but also on experienced surgical technique and early postoperative rehabilitation [25].

Currently, there is no consensus on the perioperative management of vWD patients. However, for vWF type II, some authors argue that these patients generally need multiple doses of vWF concentrates post-surgery. Emerging research indicates that recombinant vWF (rVWF) might offer a longer half-life compared to plasma derived vWF (pdVWF) concentrates, possibly due to its different glycosylation profile. Additionally, rVWF appears to enhance the stabilization of endogenous factor VIII, which may reduce the frequency of dosing needed compared to pdVWF concentrates. In one case, the patient was treated with 45 IU/kg of pdVWF concentrate preoperatively, achieving plasma levels of vWF-to-Ag 128 IU/dL, vWF-to-RCo 102 IU/dL, and FVIII-to-C 125 IU/dL. Postoperatively, levels showed a decline, necessitating an additional 25 IU/kg of vWF concentrate 12 h after surgery. Still, more research is required to understand the clinical relevance and whether this treatment is effective [26].

The management of thrombosis is also a critical consideration in vWF patients. Despite the low incidence of venous thromboembolism (VTE) in vWD patients receiving vWF replacement, the risk is not negligible, particularly in patients with additional risk factors such as obesity, advanced age, or estrogen use. Elevated plasma FVIII: C levels are known risk factors for VTE, prompting guidelines to suggest keeping these levels under specific thresholds postoperatively. Thromboprophylaxis should be considered once adequate hemostatic control is achieved, using measures such as thromboembolic deterrent stockings, and encouraging early mobilization [27–29].

Although simBTKR is a procedure associated with notable economic benefits and reduced costs, particularly in the general population, it is essential to underscore the significant risks posed by this approach, especially in patients with vWD. Unlike the general population, where simBTKR is considered a viable option, the landscape changes when it comes to individuals with vWD. This bleeding disorder introduces a heightened susceptibility to bleeding complications, rendering simBTKR a risky endeavor. The inherent challenges in managing hemostasis in vWD patients during and after surgery necessitate a cautious approach, to decrease the probability of severe complications like hematomas, hemarthroses, and even vascular injuries. However, our approach included PST, which decreases the time needed to complete the simBTKR and avoids the use of intramedullary rods.

Several limitations should be acknowledged in this case report. Firstly, the report presents findings from a single patient, limiting generalizability to broader populations with vWD undergoing BTKR. Moreover, we advocate for simBTKR using PST when we have not tried this in our case due to resource constraints. Thirdly, vWD encompasses a spectrum of subtypes with varying degrees of severity and bleeding tendencies. The management approach described in this report may not be universally applicable to all vWD patients, particularly those with different subtypes or comorbidities. This also relates to the inability to verify the patient’s diagnosis since the confirmatory diagnosis was carried out in another institution with lost documentation. Due to financial constraints, advanced perioperative hemostatic monitoring, such as vWF-to-RCo or Factor VIII level assessments, could not be performed. Additionally, while a 24-month follow-up may be considered short-term by orthopedic surgeons, it is deemed sufficient in this case to evaluate immediate complications, particularly the risk of bleeding. Lastly, although the report outlines a hematological protocol for managing vWD during the perioperative period, detailed laboratory parameters and monitoring intervals are not extensively discussed in our case due to financial constraints.

Finally, ongoing research into perioperative management strategies and emerging treatments, such as recombinant rVWF, holds promise for further optimizing outcomes in vWD patients undergoing TKR [26].

## Conclusion

The case presented highlights the successful management of BTKR in a patient with vWD Type 3. Due to limited resources, a sequential approach to TKR using PST was undertaken. The patient’s hematological protocol, including tranexamic acid administration and clotting factor replacement therapy, played a pivotal role in controlling bleeding during and after surgery. Close monitoring and management of postoperative hemoglobin levels, coupled with structured physiotherapy, facilitated successful rehabilitation and restoration of knee functionality. Furthermore, the case underscores the importance of individualized treatment plans and comprehensive perioperative management in vWD patients undergoing major surgical interventions. While simBTKR may offer economic benefits, the heightened bleeding risks associated with vWD necessitate a cautious approach and consideration of alternative strategies, such as sequential surgery and advanced surgical techniques like PST.

## Electronic supplementary material

Below is the link to the electronic supplementary material.

Supplementary Material 1

## Data Availability

Due to patient confidentiality concerns, further information or images may be requested from the first author upon request.
