# Supplementary material for: Bilateral total knee replacement for hemophilic arthropathy: a case report
Source: BMC Musculoskelet Disord. 2025 Apr 25;26:417. doi: 10.1186/s12891-025-08611-1 (PMC12023443; doi:10.1186/s12891-025-08611-1)
Supplement: Supplementary file 1 — Supplementary Material 1 [file 12891_2025_8611_MOESM1_ESM.pdf]

CARE case report guidelines

CARE Checklist of information to include when writing a case report

(cc) BY-NC-ND

Topic	Item	Checklist item description	Reported on Line
Title	1	The diagnosis or intervention of primary focus followed by the words "case report"	1-2
Key Words	2	2 to 5 key words that identify diagnoses or interventions in this case report, including "case report"	20-21
Abstract (no references)	3a	Introduction: What is unique about this case and what does it add to the scientific literature?	45-48
	3b	Main symptoms and/or important clinical findings	48-56
	3c	The main diagnoses, therapeutic interventions, and outcomes	56-58
	3d	Conclusion—What is the main "take-away" lesson(s) from this case?	58-60
Introduction	4	One or two paragraphs summarizing why this case is unique (**may include references**)	64-88
Patient Information	5a	De-identified patient specific information.	90
	5b	Primary concerns and symptoms of the patient.	90-94
	5c	Medical, family, and psycho-social history including relevant genetic information	94-98
	5d	Relevant past interventions with outcomes	94-98
Clinical Findings	6	Describe significant physical examination (PE) and important clinical findings.	90-98
Timeline	7	Historical and current information from this episode of care organized as a timeline	NA
Diagnostic Assessment	8a	Diagnostic testing (such as PE, laboratory testing, imaging, surveys).	116-118
	8b	Diagnostic challenges (such as access to testing, financial, or cultural)	116-118
	8c	Diagnosis (including other diagnoses considered)	99-100
	8d	Prognosis (such as staging in oncology) where applicable	NA
Therapeutic Intervention	9a	Types of therapeutic intervention (such as pharmacologic, surgical, preventive, self-care)	115
	9b	Administration of therapeutic intervention (such as dosage, strength, duration)	116-142
	9c	Changes in therapeutic intervention (with rationale)	116-142
Follow-up and Outcomes	10a	Clinician and patient-assessed outcomes (if available)	144-150
	10b	Important follow-up diagnostic and other test results	NA
	10c	Intervention adherence and tolerability (How was this assessed?)	NA
	10d	Adverse and unanticipated events	NA
Discussion	11a	A scientific discussion of the strengths AND limitations associated with this case report	161-224
	11b	Discussion of the relevant medical literature **with references**.	161-224
	11c	The scientific rationale for any conclusions (including assessment of possible causes)	225-240
	11d	The primary "take-away" lessons of this case report (without references) in a one paragraph conclusion	234-240
Patient Perspective	12	The patient should share their perspective in one to two paragraphs on the treatment(s) they received	NA
Informed Consent	13	Did the patient give informed consent? Please provide if requested	Yes [X] No [ ]
